# Evaluation of *sxtA* and rDNA qPCR assays through monitoring of an inshore bloom of *Alexandrium catenella* Group 1

**DOI:** 10.1038/s41598-019-51074-3

**Published:** 2019-10-10

**Authors:** Shauna A. Murray, Rendy Ruvindy, Gurjeet S. Kohli, Donald M. Anderson, Michael L. Brosnahan

**Affiliations:** 1 Climate Change Cluster, University of Technology Sydney, Ultimo, NSW 2007 Australia; 2 Woods Hole Oceanographic Institution, MS # 32, 266 Woods Hole Road, Woods Hole, Massachusetts, 02543 United States; 3 Present Address: Ramaciotti Centre for Gene Function Analysis, University of New South Wales, Sydney, NSW 2052 Australia

**Keywords:** Microbial ecology, Marine biology

## Abstract

*Alexandrium catenella* (formerly *A. tamarense* Group 1, or *A. fundyense*) is the leading cause of Paralytic Shellfish Poisoning in North and South America, Europe, Africa, Australia and Asia. The quantification of *A.catenella* via *sxtA*, a gene involved in Paralytic Shellfish Toxin synthesis, may be a promising approach, but has not been evaluated *in situ* on blooms of *A. catenella*, in which cell abundances may vary from not detectable to in the order of 10^6^ cells L^−1^. In this study, we compared *sxtA* assay performance to a qPCR assay targeted to a species-specific region of ribosomal DNA (rDNA) and an established fluorescent *in situ* hybridization (FISH) microscopy method. Passing-Bablok regression analyses revealed the *sxtA* assay to overestimate abundances when <5 cell equivalents *A. catenella* DNA were analysed, but otherwise was closer to microscopy estimates than the rDNA assay, which overestimated abundance across the full range of concentrations analysed, indicative of a copy number difference between the bloom population and a culture used for assay calibration *a priori*. In contrast, the *sxtA* assay performed more consistently, indicating less copy number variation. The *sxtA* assay was generally reliable, fast and effective in quantifying *A. catenella* and was predictive of PST contamination of shellfish.

## Introduction

Saxitoxin (STX) and its many analogues, known as the paralytic shellfish toxins (PSTs), are neurotoxins that can have profound effects on marine food chains^1^ and are responsible for causing the syndrome paralytic shellfish poisoning (PSP) in humans. In marine environments, STX-producing phytoplankton comprise ~8 species of the genus *Alexandrium*, as well as *Gymnodinium catenatum* and *Pyrodinium bahamense*^2–6^.

Because these blooms threaten seafood safety, they are monitored extensively through a variety of frequently labour-intensive methods (reviewed in^7^). Quantitative polymerase chain reaction (qPCR) based methods are a compelling alternative to more commonly applied microscopy-based approaches because they are species specific and can be low-cost, especially when integrated with automated DNA extraction and multiplexed with other, similar PCR-based water quality assays. Many previous studies have developed and applied qPCR assays for detection of *Alexandrium* sp. ribosomal RNA sequences in seawater extracts, targeting *A. catenella* (=*A. fundyense*, *A. tamarense* Group I), *A. pacificum* (=*A. tamarense* Group IV), *A. minutum*, *A. ostenfeldii*, and *A. tamutum* (e.g.,^8–22^). The amplicon targets of these assays have high copy numbers (i.e., commonly >10^5^ copies cell^−1^). None of these assays, however, is able to quantify all PST producing species.

In recent years, the identification of the core genes responsible for the PST biosynthesis pathway in *Alexandrium* spp., *Pyrodinium bahamense* and *Gymnodinium catenatum*^23–25^ have enabled the detection of genes involved in PST biosynthesis from environmental samples^26^. A group of eight genes (*sxtA, sxtB, sxtD, sxtG, sxtH or sxtT*, *sxtI, sxtS*, and *sxtU*) may be directly implicated in PST synthesis in dinoflagellates^27,28^, in particular the synthesis of the parent compound, STX, which is hypothesised to subsequently be modified to form the other PST analogs. *sxtA* is considered to be the first gene in the PST biosynthetic pathway^27,29^. *sxtA* has four catalytic domains, and shows two isoforms: one of which comprises four domains, *sxtA1–sxtA4*, while the other one encompasses only the domains *sxtA1–sxtA3*^29^, and may represent a duplicated, paralogous copy of *sxtA1*. The latter *sxtA1-A3* isoform is present in several species of dinoflagellates that do not produce PSTs^30^. The domain *sxtA4*, however, appears to be restricted to PST producing species, and has now been found with high sequence conservation in 40 strains (35 *Alexandrium*, 1 *Pyrodinium*, 4 *Gymnodinium*) of 8 species of STX-producing dinoflagellates, and is absent from comprehensive transcriptomic libraries of 67 strains of 54 species of non STX-producing species, representing different dinoflagellate families^25^.

There are several potential advantages to the use of a quantitative assay based on the detection of a gene related to toxicity, rather than detecting individual species. Firstly, all potential PST-producing species of *Alexandrium, Pyrodinium bahamense* and *Gymnodinium catenatum* can be counted in a single assay, without detecting non-STX producing species which may be important in regions where closely related toxic and non-toxic *Alexandrium* species co-occur (ie^31–35^). During the last 20 years, there have been several incidences when a new PST-producing species has been detected that was not previously known to occur in that region^36,37^. Therefore an assay to detect all PST-producing species would reduce the risk that such an occurrence might be overlooked using species-specific assays. Additionally, there is some evidence that the genomic copy number of *sxtA* within species may be more consistent than that of ribosomal DNA loci, which can vary considerably in some species of *Alexandrium*^14,26,38^. It has also been shown that, at least within the species *Alexandrium minutum*, the copy number of *sxtA4* may be correlated to the amount of PSTs produced different strains^39^.

Regions of the gene *sxtA*, generally the *sxtA4* domain, have now been successfully detected and quantified in seawater samples as a method for PST detection, during blooms of *A. pacificum* in Australia^40^, New Zealand^30^, and China^41^, *A minutum* and *A. ostenfeldii* in Norway^23^, *Gymnodinium catenatum* in Spain^23^, *A. ostenfeldii* in Finland^42^, and *A. minutum* in the Mediterranean^43^.The *sxtA4* domain has also been detected and quantified from the digestive gland of Pacific oysters (*Crassostrea gigas*) that had been experimentally fed *A. minutum*^44^, showing that this method can be used to detect the potential presence of PSP toxins in the stomach contents of commercial oysters^44^.

*Alexandrium catenella* (previously *A.tamarense* or *A. fundyense* Group I, (see^44–46^) is a leading cause of PST contamination in several areas of the world, including North and South America, China, the UK and recently, Australia^47–50^. As one example of this species’ impact, a 2012 bloom in Tasmania, Australia, cost the region ~$AUD 23 million^51^. In North and South America, *A. catenella* is the most common PST-producing species. Blooms along the east and west coasts of the U.S. and Canada cause annual closures of recreational and commercial shellfish harvesting and require extensive monitoring programs^6^. Similar blooms in the southern hemisphere have been recorded since 1980 in Argentina, and more recently in Brazil^52^. Closely related species within the former *Alexandrium tamarense* species complex, particularly *A. pacificum* (formerly, *A. catenella*, Group IV), frequently cause shellfish harvesting closures due to PST detection in shellfish in more temperate and subtropical waters of the western Pacific, for example, in New Zealand^35^, Australia^26^, Japan^15^, Korea, and China^34^.

To date, no study has examined the efficacy of the use of an assay based on *sxt* genes for the detection and quantification of *Alexandrium catenella* cell abundance during blooms. While molecular genetic assays have been applied as tools to quantify the abundance of HAB species for many years, validation of these assays has often failed to demonstrate equivalence with standard microscopy-based approaches^8–22^. This is particularly true of qPCR assays targeting dinoflagellates which sometimes vary in the copy number of the ribosomal loci that these assays most commonly target^8–22^. Many field validation efforts have also suffered from limited sampling that explores only a relatively small range of cell concentrations and does not assess performance versus different life cycle stages. The aim of this study was to compare abundance estimates of *Alexandrium catenella* from qPCR assays targeting LSU rDNA and *sxtA4* to abundance estimates from a well-established, FISH microscopy-based approach, and to thoroughly validate these approaches.

## Materials and Methods

### Sample collection

**Figure 1:**
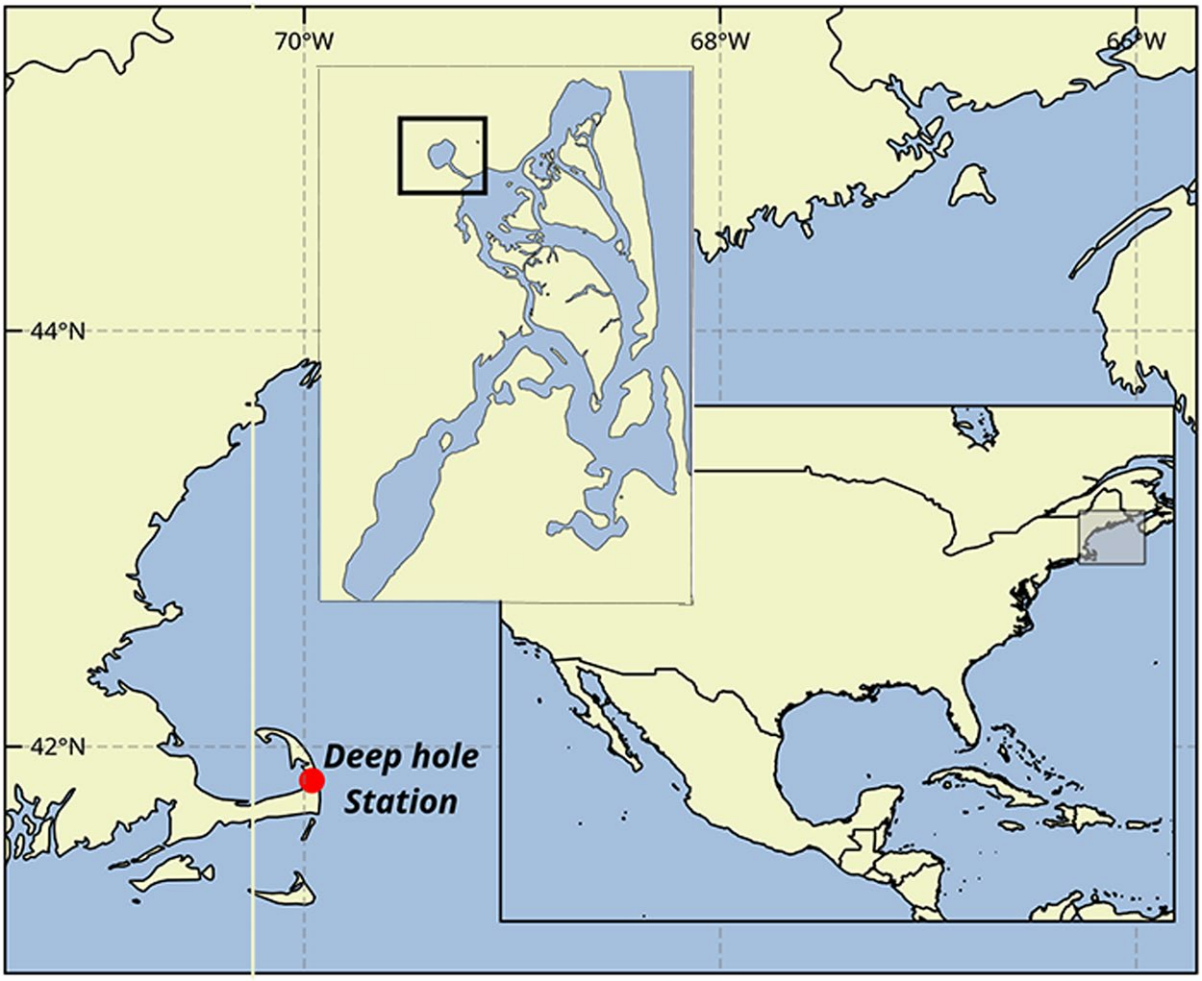
Map of the sampling location, Salt Pond, MA, USA. The inset box shows the Nauset Marsh system.

Salt Pond (Eastham, MA USA) (Fig. 1) has been the site of ongoing field investigations of *A. catenella* because its bathymetry and stratification properties, combined with the vertical migration behaviour of the *A. catenella* cells, lead to the localization of this species within the confines of the pond. As a result, the progression of blooms at the site can be tracked without consideration of advection and patchiness that complicate similar efforts in more open coastal areas. Selective retention of *A. catenella* also causes blooms to routine exceed concentrations of 10^5^ cells L^−1^, enabling efficient testing of assays across the full range of cell concentrations that might be encountered in bloom monitoring efforts^53–55^. The pond is approximately 320 m in diameter and has a maximum depth of 9 m near its centre. Samples were collected approximately weekly from 4^th^ March – 22^nd^ May 2013 and in triplicate with a 5 L Niskin bottle from 1 and 5 m depths over the deep central area of the pond. From each bottle, a 4 L primary sample was concentrated over 20 µm Nitex mesh, then resuspended in 20 mL of <15 µm filtered seawater. The concentrate was then split in equal parts (equivalent to 2 L raw seawater) for analysis by qPCR and the other for counting using a microscopy-based protocol that positively identifies *A. catenella* through staining with a species-specific oligonucleotide probe^8^. No other PST-producing species have been recorded in over three decades of phytoplankton investigations at this site.

### Microscopy-based identification and enumeration

All microscopy-based counts of field material employed a well-established fluorescent *in situ* hybridization (FISH) protocol that positively identifies *A. catenella* cells through staining with a species-specific ribosomal RNA (rRNA) probe. Microscopy sample concentrates were fixed with 5% unbuffered formalin in the field then stored on ice for transport to the Woods Hole Oceanographic Institution (Woods Hole, MA USA). There, cells were immediately pelleted by centrifugation (3,000 rcg, 10 minutes) and resuspended in ice cold methanol for storage at −20 °C until staining with Cy3-labeled NA1 probe (5′-AGT GCA ACA CTC CCA CCA-3′)^8^. In preparing slides for counting, up to half the volume of the methanol suspension (equivalent to 1 L of raw seawater) or approximately 400 cells were applied to a 5.0 µm Durapore filter for staining, whichever was a lesser volume of the methanol suspension. When the estimated suspension volumes were less than 1 mL, concentrates were diluted through a series of 10-fold serial dilutions with fresh methanol as needed to apply approximately 400 cells to filters in a volume of 1–7 mL of methanol. After staining, filters were mounted on slides and all *A. catenella* were counted. For most samples, the suspension volume applied was estimated from comparisons to samples collected earlier in the bloom. This led to some filters having >2500 cells or <100 cells. In these cases, samples were re-prepared using dilutions that represented no more than half of the original methanol suspension (equivalent to 1 L of the original seawater collection).

### Culture maintenance

*Alexandrium catenella* strain ATMP7E8 was isolated from Salt Pond. Cultures for use as standards were grown at 18 °C with a 12/12-h light:dark photoregime at 60 mm^2^ s^−1^. *Alexandrium* cultures were grown in GSe medium and harvested during late exponential phase. Two samples of this strain were extracted for DNA analysis to determine LSU and *sxtA4* copy numbers of this strain.

### Toxin determination

Samples of *Alexandrium catenella* strain ATMP7E8 were extracted using the method of Harwood *et al*.^56^. Briefly, 1 mM acetic acid was added and vortexed for 90 sec, before samples were placed in a water bath at 100 degrees for 5 min. After cooling, samples were centrifuged and filtered with a PVDF filter (0.45 uM). The supernatant was used with or without dilution for LC-MS analysis.

A Thermo Scientific™ Q EXACTIVE™ high resolution mass-spectrometer equipped with an electrospray ionization source was used for the detection of biotoxins. The following source parameters were used in all experiments: a capillary temperature of 263° C, a spray voltage of 3.5 kV, an auxiliary gas heater temperature of 425° C, a sheath gas and an auxiliary gas flow rate of 50 and 13 (arbitrary units). The mass spectrometer was operated both in positive and negative ion mode scanning across the range of m/z 100–500. For detection of PST toxins, a target-mass of each toxin [M + H] + or [M − H]- was extracted with a mass tolerance of 5 ppm. For quantitation, peak areas were integrated and sample concentrations calculated from linear calibration curves generated from external standards. Thermo Xcalibur (version 3.0.63) and TraceFinder EFS (version 3.2) softwares (Thermo Fisher Scientific, Inc.) were used for data analysis.

Chromatographic separation was performed on a Thermo Scientific™ ACCELA™ UPLC system. Analysis was performed using an Acquity UPLC BEH Phenyl 1.7 µm 100 × 2.1 mm column with an injection volume of 5 µL. The mobile phases used were A1 (water/formic acid/NH4OH at 500:0.075:0.3 v/v/v), B1 (acetonitrile/water/formic acid at 700:300:0.1 v/v/v), A2 (water/formic acid at 200:1 v/v) and B2 (methanol). Initial condition starts at A:B 5:95 at a flow rate of 400 µl/min and held for 4.0 min. The condition was then linearly changed over 3.5 min from A:B (5:95) to A:B (50:50). The flow rate was then changed from 400 µl/min to 500 µl/min over 2.0 min. The chromatographic condition was then rapidly changed to initial buffer conditions A:B (5:95) over 0.3 min while the flow rate was kept constant. The flow rate was then increased to 650 µl/min over 0.2 min and held for 0.6 min. The flow rate was then decreased back to initial flow rate 400 µl/min and re-equilibrated for 2.3 min before injecting next sample. Certified standard solutions of C1,C2, GTX1, GTX2, GTX3, GTX4, GTX5, dcGTX2, dcGTX3, STX, dcSTX, NEO and dcNEO were purchased from National Research Council of Canada (NRC, Halifax, Nova Scotia, Canada).

### Mouse bioassay toxin analysis

Mussels were collected weekly for PST analysis at a shallow site (1–1.5 m deep at high tide) on the western shore of Salt Pond during the months of April and May. Mouse bioassays were conducted on pooled shellfish samples using the AOAC method^57^ by the Massachusetts Department of Marine Fisheries (https://www.mass.gov/service-details/psp-red-tide-monitoring).

### DNA extraction and PCR methods

Phytoplankton samples were extracted with the FastDNA Spin kit for soil (MP Biomedicals), according to the supplied protocol. Sample filters on which large amounts of biomass was visible were dissected into two using a scalpel, and DNA was extracted separately on each half. DNA was eluted from binding matrix with 200 µl of water. When extracted in two fractions, DNA was combined after elution. All DNAs were then vacuum dried at 55 °C (Concentrator plus, Eppendorf) and resuspended in a final volume of 40 µl. The final qPCR assay used 1 µl per replicate. DNA quality and quantity were determined using a Nanodrop (Thermoscientific). DNA concentrations of samples were in the range of 5–90 ng/µl.

Each qPCR assay had a final volume of 20 µL, and comprised 10 µL of Sybr Select Master Mix (Life Technologies), which contains Sybr green dye, Amplitaq DNA polymerase with a hot start mechanism, ROX passive dye reference, heat labile Uracil DNA glycolase (UDG), dNTP blend and buffer; 1 µL of template DNA, representing 2.5% of the total sample DNA, and 1 µL of 0.5 µM primers. Assays were performed in triplicate under the following run conditions: 95 °C for 10 min, 40 × 95 °C for 15 s and 60 °C for 30 s. To confirm the specificity of each amplification, melt curve analysis was conducted at the end of each run by increasing the temperature to 95 °C for 15 s, 60 °C for 1 min, and 95 °C for 15 s. qPCR analysis was carried out with a Step One Plus (Life Technologies).

The primers used targeted *sxtA4*^40^ and the D1-D2 region of LSU rDNA of *Alexandrium catenella*^18^, and were: *sxtA4*: Forward 5′ CTGAGCAAGGCGTTCAATTC ′3, Reverse 5′ TACAGATMGGCCCTGTGARC ′3; *A. catenella*: Forward 5′ GGCATTGGAATGCAAAGTGGGTGG ′3, Reverse 5′GCAAGTGCAACACTCCCACCAAGCAA ′5.

Standard curves of *sxtA4* and the D1-D2 rDNA of *A.catenella* were derived from DNA extracted from 3–4 replicate pellets of known cell numbers. For *sxtA*, aliquots of DNA were diluted at a ratio of 1:2 for 6 successive dilutions, while for LSU, aliquots were diluted at a ratio of 1:10 for 6 successive dilutions. The standard curve was used to determine the approximate equivalent number of *A. catenella* “cells” that could be detected. To assess the presence of inhibition in DNA of samples, a standard curve was conducted using a 5 fold dilution series of a 1:10 dilution of a sample DNA.

In order to determine the genomic copy number of the gene *sxtA4* in the DNA extracts from strain ATMP7E8, a standard curve was developed by using known concentrations of *sxtA4* sequences (Gblock, IDT). DNA extracts from known numbers of ATMP7E8 cells were then analysed by qPCR in triplicate for comparison to the *sxtA4* standards.

### Statistical analyses

Passing-Bablok (PB) regressions were used to assess agreement between the two qPCR assays and the microscopy-based estimates of cell concentration over the range observed during the bloom (from 0 to 355250 cells L^−1^ as measured by microscopy). PB regression is a robust, nonparametric method for comparing measurement methods that is tolerant of error in both measurements^58^. Confidence intervals associated with slope and intercept estimates indicate whether these parameters are significantly different from 1 and 0, respectively. Linearity of relationships between measurement methods is tested by cusum^58^. In these analyses and all other statistical tests, concentration estimates were log-transformed for homogeneity of variance across the measured sample range. Microscopy was considered an unbiased reference method, albeit one with higher relative uncertainties at low cell concentrations because estimates are derived from lower total numbers of cells counted^59^. Regression residuals were also inspected across the range of observed sample concentrations and by sampling date to assess whether disagreement between methods might be attributed to copy number changes or changes in the abundance of extracellular target DNAs (e.g., with increasing abundance of grazer species that may have consumed *A. catenella* cells).

Regression relationships were refined in two ways. First, for the LSU assay, estimates of cell abundance were recalibrated using microscopy-derived estimates from the bloom’s development period. Both qPCR assays provide estimates of the number of amplicon targets present in DNA extracts and cell abundance is derived from an estimate of the target copy number cell^−1^. Discrepancies in the copy number of ribosomal DNA are widely reported (e.g.,^14,38^) and therefore a choice of any single clone as a standard is somewhat arbitrary. Difference in the copy number estimate derived from the ATMP7E8 culture and the Salt Pond population was therefore determined via linear regression of qPCR-derived estimates to microscopy-based ones with intercept set to 0, e.g.,

$${A}_{M}=C\times {A}_{qPCR}$$

where *A*_*M*_ and *A*_*qPCR*_ are abundances estimated by microscopy and qPCR, respectively, and *C* is a conversion factor describing the proportional difference in copy number between the ATMP7E8 culture and the Salt Pond population. The difference in copy number was far larger for the LSU target than for *SxtA*, and therefore only the LSU-derived estimates were corrected by the estimate of *C* determined through this analysis.

The second refinement explored was developed through a Bland-Altman comparison of the two qPCR assays. Bland-Altman plots show error magnitude and direction across the range of measured quantities, revealing quantity-dependent errors that can be masked by simple comparisons of sample means. Discrepancies between equivalent measurement methods are expected to have a mean of 0 across the full range of quantities to be measured^60^. The qPCR approach is particularly amenable to this type of comparison because DNA extracts could be analysed in replicate by both assays. Because the comparison revealed a threshold concentration below which the *sxtA* assay could detect but not accurately quantify its target, PB regression was repeated using only the subset of samples whose concentrations exceeded this threshold.

## Results

### Efficiency and sensitivity of qPCR Assays

The qPCR assay based on *sxtA4* was found to be 93–94% efficient when tested using *A.catenella* ATMP7E8. The number of genomic copies of *sxtA* per cell in two separate DNA extractions of the *A. catenella* strain from Salt Pond was found to be 245 +/− 30 and 325 +/− 68 copies cell ^−1^ and the assay successfully detected *sxtA* standards at levels less than 3 cell equivalents in individual reactions.

**Figure 2:**
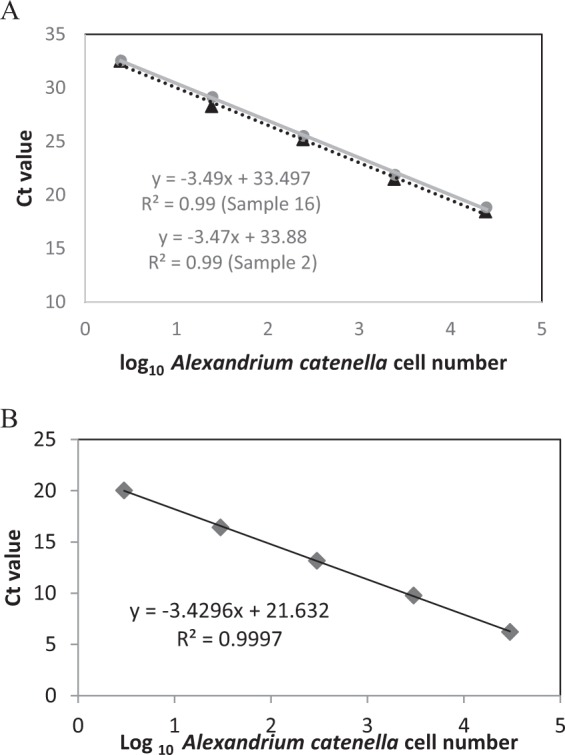
Standard curve of *sxtA* (**A**) and LSU rRNA (**B**) using DNA extractions of *A. catenella*, strain ATMP7E8 isolated from Salt Pond, showing efficiency (%), dilution series, (+/− error bars are standard deviations).

Efficiency was slightly higher for the LSU rRNA qPCR assay (96%) when tested versus extracts of *A. catenella* ATMP7E8 (Fig. 2) and the assay could detect cells at levels less than 0.005 cell equivalents reaction^−1^. A 5-fold dilution series of a dense sample also showed that inhibition was negligible (slope −3.13, efficiency = 108.7%) (Supplementary Fig. 1). PCR inhibition was therefore unlikely to have influenced amplification results of the environmental samples.

### Toxins

*A. catenella* ATMP7E8 from Salt Pond was found to contain C toxins as the major component of the toxin profile (Table 1), with the analogs C1. C2 as the most common components. The strain also produced small quantities of GTX1, dcGTX3, and NEO STX. Trace quantities were found of GTX2, dc GTX2, GTX4, GTX5, STX.

**Table 1:** Concentration of PST toxins in strain ATMP7E8 from Salt Pond.

Target Compounds	Amount (pg cell^−1^)
C1	11.05
C2	3.72
GTX2	0.23
GTX1	2.76
dcGTX2	0.03
GTX3	0.00
GTX4	0.20
dcGTX3	1.41
GTX5	0.03
dcNEO	0.00
STX	0.06
dcSTX	0.00
NEO	0.52
Total	20.01

### Comparison of FISH- and LSU and *sxtA4* qPCR-based estimates of cell concentration

**Figure 3:**
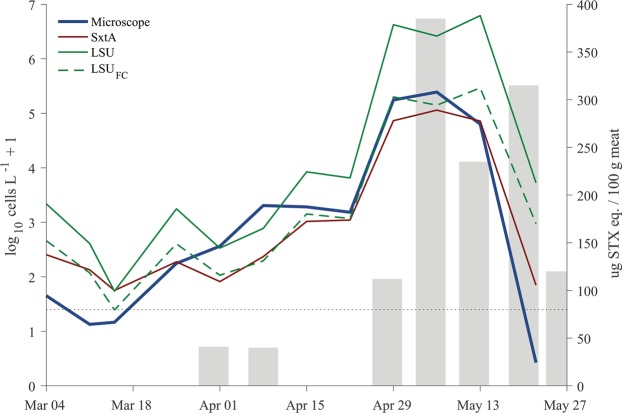
Mean of cell concentration estimates from samples collected at 5 m by survey day through the full course of the 2013 *A. catenella* bloom in Salt Pond. LSU_FC_ data are revised estimates of cell concentration based on comparison to microscopy counts as described in the text. Gray bars indicate PST levels measured by mouse bioassay in *Mytelis edulis* collected from Salt Pond by the Massachusetts Department of Marine Fisheries (limit of detection: 40 µg STX eq./100 g shellfish meat). Linear correlations between PST levels and cell concentrations from 7 days previously as measured by microscopy and *sxtA* (n = 8) were highly significant (r^2^ = 0.93, 0.91, respectively, p < 0.0001 for both).

A total of 72 paired samples were collected over the course of the 2013 bloom in Salt Pond, from its early development through termination. Cell abundance increased steadily through the month of April and peak concentrations were observed May 6 (Fig. 3). Cell concentrations at 5 m were 1–2 orders of magnitude higher than at 1 m until the final sample collection (May 22). These observations are consistent with a previously published study that described a concerted sexual transition by the 2013 bloom on May 9, after which planozygotes cells altered their diel vertical migration, swimming nearer to the surface during the day^61^.

Results from all three enumeration methods were strongly correlated with one another and each captured the bloom’s development and decline. *A. catenella* were detected in all 72 sample extractions by the two assays while the FISH method failed to detect cells from 5 of the bottle collections.

Initial PB analyses of results from the qPCR methods revealed relationships that were statistically different than 1:1 correspondence with FISH microscope counts (Table 2). The *sxtA* assay overestimated cell concentrations <100 cells L^−1^ while the LSU assay overestimated cell concentrations across the full range tested. The latter result indicated the ATMP7E8 culture had a 24.3% higher LSU copy number than the Salt Pond population. This error was corrected by calibrating LSU results with FISH microscope counts from samples collected during bloom development (4 March–6 May). Estimates from 13 and 22 May samples (bloom decline) were omitted because DNA from grazed cells was expected to make a larger contribution to the observed qPCR signals in these samples (e.g., Haley *et al*. 2013) and because copy number may vary among sexual life cycle stages that predominated during the bloom’s decline^38,61^. Revised LSU qPCR estimates more closely matched estimates from FISH microscopy and produced estimates of slope and intercept close to 1 and 0 in PB regression (Table 2).

**Table 2:** Results from Passing-Bablok regression of qPCR and microscopy measurements of *A. catenella* concentrations during the 2013 bloom in Salt Pond (Eastham, MA USA).

Method Comparison	N	Slope (95% CI)	Intercept (95% CI)	Cusum
*sxtA*, microscopy	72	0.71 (0.59–0.85)	0.96 (0.59–1.21)	P > 0.1
*sxtA*, microscopy (≥100 cells L^−1^)	36	1.09 (0.92–1.24)	−0.56 (−1.07–0.001)	NA
LSU, microscopy	72	1.21 (1.08–1.38)	0.06 (−0.23–0.46)	P > 0.1
LSU_FC_, microscopy	72	0.96 (0.85–1.08)	0.11 (−0.11–0.40)	P > 0.1

P-value from cusum tests are indicated when sample size is greater than 50. Nonsignificant cusum results indicate a failure to reject a linear relationship.

**Figure 4:**
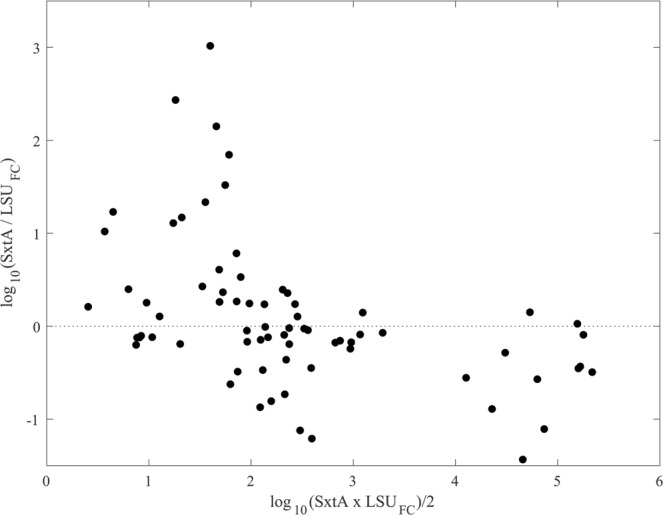
Bland Altman plot comparing log-transformed *sxtA* and field-calibrated LSU assay derived estimates of *A. catenella* abundance. Points are means from triplicate qPCR assays of a single DNA extract. The *sxtA* assay significantly overestimated cell concentration in samples with fewer than 200 cells, a threshold that corresponds to about 5 cell equivalents or approximately 10^3^ *sxtA* copies per reaction. Were assays equivalent points would be tightly clustered about 0 across the full range of measured concentrations (x-axis).

Bland-Altman comparison of qPCR measurements showed a clear transition in the behaviour of the *sxtA* assay at a threshold of 100 cells L^−1^ (corresponding to 5 cell equivalents reaction^−1^). The *sxtA* assay estimates were on average nearly an order of magnitude lower than the LSU qPCR results above this threshold and much greater below it (Fig. 4). Standard deviation between the LSU and *SxtA* results from samples above the threshold was similarly large, nearly 1.5 orders of magnitude above the mean estimate from any given sample. Comparison of *sxtA* to microscopy-derived estimates ≥100 cells L^−1^ indicated a small difference in copy number (6.3% fewer copies in the ATMP7E8 culture). Repeat PB analysis of this smaller subset of the data produced estimates of slope and intercept that were statistically consistent with a 1:1 relationship between *sxtA* assay results and FISH microscopy counts (Table 2).

**Figure 5:**
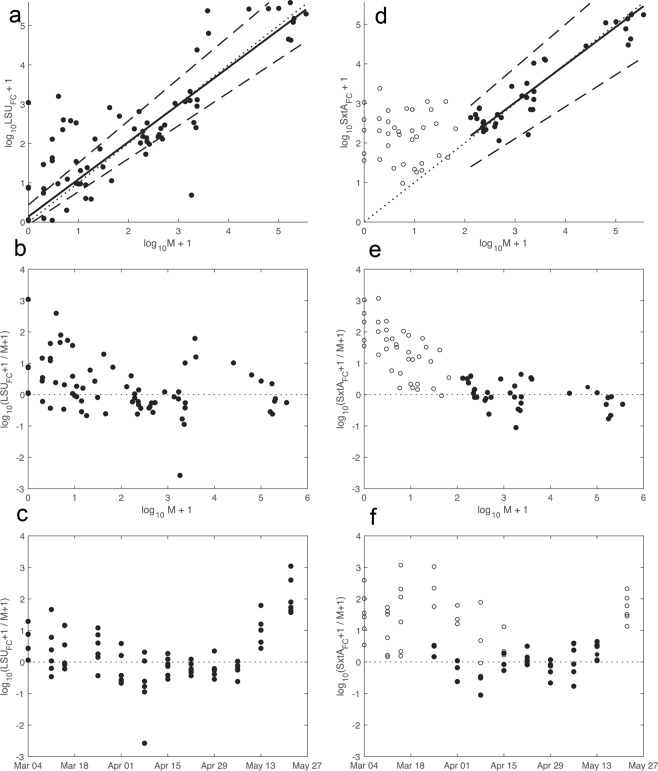
Passing Bablok regression and residual analyses. (**A**) Results from regression of microscopy and field-calibrated, LSU qPCR estimates of cell concentration. Solid line is the linear relationship described by parameters in Table 2, dotted line is 1:1, and dashed lines are upper and lower bounds from 95% confidence interval estimates of the regression. (**B**) Log-transformed residuals from the LSU-microscopy regression across the range of concentrations as estimated by microscopy. (**C**) The same log-transformed residuals but plotted by sampling day. The LSU assay tended to overestimate cell concentrations during early bloom development (March 4 and 11) and bloom termination (May 13 and 22). (**D**) Regression of microscopy and field-calibrated, *sxtA* qPCR estimates of cell concentration derived from the subset of samples ≥100 cells L^−1^ by microscopy (closed, black circles). Open circles are data points excluded from the regression (microscopy estimate <100 cells L^−1^). (**E**) Log-transformed residuals from the *sxtA*-microscopy regression across the range of observed cell concentrations. The *sxtA* assay increasingly overestimated cell concentrations in samples below the 100 cells L^−1^ threshold. (**F**) Log-tranformed residuals from *sxtA*-microscopy regression plotted by sampling day. Samples with cell concentrations >100 cells L^−1^ (closed, black circles) are more tightly clustered about 0 (no error) than samples with lower concentrations (open circles) and as estimated using the LSU assay (left column).

Inspection of PB residuals (i.e., the differences between paired microscopy and qPCR estimates) revealed similar patterns of error by the two qPCR assays (Fig. 5). Both tended to overestimate cell concentrations early during the bloom’s development and throughout bloom termination. However, severe overestimation errors (>1 order of magnitude) were restricted to samples with fewer than 200 cells, and then only in results from the *sxtA* assay; larger LSU errors were more evenly distributed across the sampled range of concentrations. Due to the heteroskedasticity of this data set (variance proportional to concentration), it is challenging to describe the observed variance in absolute terms. At the lower limit of the *sxtA* assay (100 cells L^−1^), standard error range was 37–273 cells L^−1^ and at the highest concentration observed (355,250 cells L^−1^) it was 129,940–971,260 cells L^−1^. At the same limits, the LSU assay’s standard error ranges were 18–546 cells L^−1^ and 65,078–1,939,300 cells L^−1^. While the LSU assay estimates were linearly correlated and unbiased across the range of concentrations examined, the estimates produced were much less precise than those from the *sxtA* assay.

### Comparison with PST amounts by mouse bioassay

In the analysis of samples for PSTs in shellfish from Salt Pond during March-May 2013, samples contained amounts of PSTs varying from <40 µg PST/100 g to 627 µg PST/100 g. PST loads in shellfish were correlated with *A. catenella* cell abundances one week prior to shellfish collections (Fig. 3).

## Discussion

Molecular genetic quantification methods for *Alexandrium* species have been developed over the past 15 years as an alternative to traditional light microscopy-based identification and quantification, and are considered rapid and reasonably precise^8–18,20,21^. Advantages identified with molecular genetic-based methods are the comparatively low limit of detection of cells, a wide dynamic range of detection, and no requirement for highly sophisticated taxonomic identification skills^8,11^. Most molecular genetic assays have used rDNA markers for detection, such as the large subunit rDNA (LSU), small SSU rDNA and 5.8 s rDNA regions, because of their high copy number and comparatively fast evolutionary rates, which enables specificity for particular genera, species and genotypes^8–10,12–14^.

In this study, abundance estimates of *A. catenella* from qPCR of either the *sxtA* gene or LSU rDNA were significantly correlated with counts using FISH microscopy but tended to produce distinct error patterns (Figs 3, 5). Both qPCR assays tended to over-estimate cell number at low abundance. A threshold concentration of 100 cells L^−1^ was identified for the *sxtA* assay in this study but the precise level of this threshold is a function of the original sample volume and the final volume of the sample DNA extract. There is significant potential to increase sensitivity of the assay through an increase in sample volume, especially given the absence of PCR inhibition observed in this sample set (Supplementary Fig. 1). Exclusion of data below this threshold was sufficient to achieve statistical similarity between *sxtA* assay and FISH microscopy-derived estimates of cell abundance (Table 2). The LSU qPCR assay tended to overestimate cell abundance across the complete range of cell concentrations sampled (Figs 3, 5), reflecting a difference in the mean LSU copy number in the field population and the ATMP7E8 culture used for calibration. Correction for this copy number difference was necessary to achieve 1:1 correspondence between the two measures. It remains to be shown whether the measured copy number of the Salt Pond population is stable year to year or is representative of other *A. catenella* populations in the region. It is also possible that copy number fluctuated over the course of the bloom. Overestimation by both assays was most prominent early in bloom development and during bloom decline, periods when ribosomal DNAs ingested by grazers may contribute a larger proportion of the total in a sample extract than during later stages of bloom development^62^. The qPCR assays also tended to overestimate abundance near their limits of detection, a pattern that is most prominent in results from the *sxtA* assay (Figs 4, 5).

Just like qPCR assays, the detection limits of microscopy-based phytoplankton methods depends on initial sample volume and sample fractionation and concentration steps. Confidence intervals associated with many count-based approaches are dependent on the number cells recorded; 400 or 100 cells providing 95% confidence intervals of 10 and 20%, respectively^63^. Additional uncertainty is introduced through successive sub-sampling steps^64^. In the case of the FISH microscopy method used here, all uncertainty is associated with subsampling rather than final counts because all cells applied to staining filters were counted (i.e., there is no uncertainty associated with ‘subsampling’ of a well slide). FISH estimates were used as a standard in this study not because they lacked error but because the approach is well-established and its errors are thought to be unbiased. Differences between the qPCR- and microscopy-derived estimates reflect the sum of the errors associated with these methods. Were qPCR assays similarly unbiased, mean difference between either qPCR method and microscopy would be 0. In fact, simple paired t-tests comparing the two qPCR assays to the microscopy estimates do detect differences (P < 0.001 in both cases). It is also the case that compensating errors across a range of concentrations can cause t-tests and similar statistical tests of mean differences to fail when measurement methods are in fact non-equivalent. Passing-Bablok regression and Bland-Altman analyses address this shortcoming by evaluating difference trends across the range of sampled cell concentrations.

Initial LSU qPCR assay estimates of cell abundance were correlated with microscopy estimates but were a little more than 25% greater on average, a result that is in line with previous ribosomal qPCR studies that have reported errors ranging from +/− 25%, at the lowest, to up to an order of magnitude^10,11^. An example is a study that found that qPCR using rDNA genes overestimated the abundance of *A. catenella* compared to the estimate using light microscopic counting by ~100%^10^. A review^11^ of 17 different methods of estimating the abundance of *A. catenella*, including 9 different microscopy-based approaches, a qPCR method based on a ribosomal RNA gene, and FISH microscopy, found that qPCR based on a ribosomal RNA gene yielded highly precise (i.e., internally consistent and reproducible) results, however, over-estimated abundance by ~300%^11^. In another recent study, an abundance estimate using a qPCR assay based on a region of rDNA was highly correlated to the abundance estimate using a cell counter for the species *A. catenella*, but the two approaches were not well correlated for the species *A. ostenfeldii*^22^. Results from the *sxtA* assay contrast with these previous reports of rDNA assays and our own results from the LSU assay, as differences in copy number of *sxtA* between the ATMP7E8 culture and the Salt Pond population appeared to be quite modest.

Most genes are present in multiple copies within dinoflagellate genomes. For example, the species *Lingulodinium polyedrum* may contain ∼30 copies of a protein kinase gene^65^, and to up to ∼5,000 copies of the mitotic cyclin gene^66^. Ribosomal RNA genes have been shown to be particularly variable in copy number between dinoflagellate species and between strains of the same species and between life cycle stages^12,14,18,38^. In studies in which the copy numbers of ribosomal RNA genes were measured in multiple strains of a species of *Alexandrium*, vegetative cells were found to differ, from 860–1020 genomic copies cell^−1^ in *A. minutum*^12^, 930–4830 genomic copies cell^−1^ in 4 strains of *A. ostenfeldii*^42^, 190,000–2,489,000 genomic copies cell^−1^ amongst 9 strains of the species *A.pacificum* (as *A. catenella*)^14^, 70,000–150,000 in *A. tamarense*, and 500,000–1,000,000 in *A. catenella*^38^. Estimates from cysts of *A. tamarense* and *A. catenella* are far lower (30,000 and 30,000–45,000, respectively, for *A. tamarense* and *A. catenella*) indicating significant reductions in copy number associated with the formation and/or maturation of sexual life cycle stages^18,38^. Copy number variations of >10-fold amongst strains of the same species may account for the variability between microscopy and rDNA qPCR assays.

The number of genomic copies of *sxtA4* has also been found to vary amongst species of *Alexandrium* studied. In studies of *A. pacificum*^26^, the number of copies in 3 strains was 180–280 genomic copies cell^−1^. A study of fifteen strains of the species *A. minutum* found that far fewer copies were present, however, the variability in copy number was somewhat greater than that in *A.pacificum* strains, 1.5–10 genomic copies cell^−1^ ^39^. In the species *Alexandrium ostenfeldii*, studies of four strains found that 3–11 genomic copies cell^−1^ of *sxtA4* were present^42^. These differences are somewhat smaller than the maximum variability seen in ribosomal RNA genes, and may account for the fact that the abundance estimates using *sxtA* qPCR generally did not differ significantly from those using light microscopy.

While discrepancies between the qPCR or FISH microscopy-based estimates are important, they must also be considered in the context of plankton patchiness within marine coastal environments and the inherent nature of errors associated with phytoplankton sampling. The same samples from each date and depth tended to show the highest variability, regardless of the abundance estimate method. Because all samples analysed in this study were paired, these errors are not reflective of patchiness in the field. Increasing variance at higher cell concentrations reported here instead reflects the underlying distribution of cells within samples, which is well approximated in many cases by a Poisson distribution (for which variance and mean are equal). Underlying heteroskedasticity is thus inherent in the microscopy-derived estimates and would be expected were paired FISH microscopy counts compared rather than counts and the results from qPCR assays.

The root cause of differences between the two qPCR assays is less clear. Replicate analyses of the same DNA extract by one or the other qPCR assay agree to within 0.1 in threshold cycle estimate. Like count-derived estimates, the effect of machine-level error (i.e., threshold cycle differences between replicates on a single qPCR plate) varies with concentration. Differences of 0.1 in estimates translate to smaller absolute differences at lower cell concentrations than at higher ones but these errors represent a small fraction of the variance observed between the two assays when applied to the same DNA extract (Fig. 4). Moreover, the effect of this type of error is mitigated by averaging of replicate qPCR analyses. Were copy number to vary among samples, one would still expect it to remain consistent among samples collected on the same day and depth. Instead, variance between methods for day/depth subsets was similar to that observed overall (and the ≥100 cells L^−1^ for *sxtA* result comparisons).

Given these inherent errors and the nature of phytoplankton distributions, no single sample or measurement is adequate if a precise estimate of cell concentration is required. Replicate sampling across the depth distribution of PST-producing organisms should be incorporated in the final design of sampling regimens. An inherent advantage of molecular methods like the qPCR assays is that they are amenable to high-throughput processing systems. Where the qPCR assays provide unbiased estimates of PST producing organisms, results from replicate samples can be averaged to provide accurate estimates of abundance near shellfish and other natural resources.

## Conclusion

Quantitative PCR based on the *sxtA* gene appears to be a reliable method for detecting PST producing *Alexandrium catenella* for samples containing >5 cell equivalents qPCR reaction^−1^. In our study, *sxtA*based abundance estimates were more tightly correlated with FISH microscopy results than a qPCR assay targeting LSU rDNA. The reasons for this require further investigation, but may relate to copy number differences in rDNA loci amongst strains and/or life-cycle stages. Cell abundance was also shown to be significantly positively correlated with the PST amounts in shellfish at the same site one week later, suggesting that cell monitoring – by *sxtA* qPCR or otherwise - can be used as an early warning system for PST contamination of shellfish.

## Supplementary information

Dataset 1

## Data Availability

The authors declare that all data is available on request to the corresponding author.
