# Supplementary material for: Evaluation of sxtA and rDNA qPCR assays through monitoring of an inshore bloom of Alexandrium catenella Group 1
Source: Sci Rep. 2019 Oct 10;9:14532. doi: 10.1038/s41598-019-51074-3 (PMC6787220; doi:10.1038/s41598-019-51074-3)
Supplement: Supplementary file 1 — Dataset 1 [file 41598_2019_51074_MOESM1_ESM.docx]

**Supplementary Data**

Evaluation of sxtA and rDNA qPCR assays through monitoring of an inshore bloom of *Alexandrium catenella* Group 1

Shauna A Murray^1^*, Rendy Ruvindy^1^, Gurjeet S. Kohli ^1,3^, Donald M. Anderson^2^, Michael L.

Brosnahan^2^

^1^Climate Change Cluster, University of Technology Sydney, Ultimo, NSW 2007, Australia

^2^Woods Hole Oceanographic Institution, MS # 32, 266 Woods Hole Road, Woods Hole, Massachusetts

02543, United States.

^3^Current Address: Ramaciotti Centre for Gene Function Analysis, University of New South Wales,

Sydney, NSW 2052, Australia

Supplementary Figure 1. Standard curve of one DNA sample (6 May, 5m depth), using LSU rDNA primers, (+/- error bars are standard deviations), showing that inhibition of the samples was relatively minor using this method (efficiency = 108.7%).
